# Fe_3_O_4_@Pt nanoparticles to enable combinational electrodynamic/chemodynamic therapy

**DOI:** 10.1186/s12951-021-00957-7

**Published:** 2021-07-10

**Authors:** Tong Chen, Qiang Chu, Mengyang Li, Gaorong Han, Xiang Li

**Affiliations:** 1 State Key Laboratory of Silicon Materials, School of Materials Science and Engineering, Zhejiang University, Hangzhou, 310027 Zhejiang China; 2 ZJU-Hangzhou Global Scientific and Technological Innovation Centre, Zhejiang University, Hangzhou, 311200 China

**Keywords:** Electrodynamic therapy, Chemodynamic therapy, GSH depletion, Fe_3_O_4_@Pt

## Abstract

Electrodynamic therapy (EDT) has recently emerged as a potential external field responsive approach for tumor treatment. While it presents a number of clear superiorities, EDT inherits the intrinsic challenges of current reactive oxygen species (ROS) based therapeutic treatments owing to the complex tumor microenvironment, including glutathione (GSH) overexpression, acidity and others. Herein for the first time, iron oxide nanoparticles are decorated using platinum nanocrystals (Fe_3_O_4_@Pt NPs) to integrate the current EDT with chemodynamic phenomenon and GSH depletion. Fe_3_O_4_@Pt NPs can effectively induce ROS generation based on the catalytic reaction on the surface of Pt nanoparticles triggered by electric field (E), and meanwhile it may catalyze intracellular H_2_O_2_ into ROS via Fenton reaction. In addition, Fe^3+^ ions released from Fe_3_O_4_@Pt NPs under the acidic condition in tumor cells consume GSH in a rapid fashion, inhibiting ROS clearance to enhance its antitumor efficacy. As a result, considerable in vitro and in vivo tumor inhibition phenomena are observed. This study has demonstrated an alternative concept of combinational therapeutic modality with superior efficacy.

**Supplementary Information:**

The online version contains supplementary material available at 10.1186/s12951-021-00957-7.

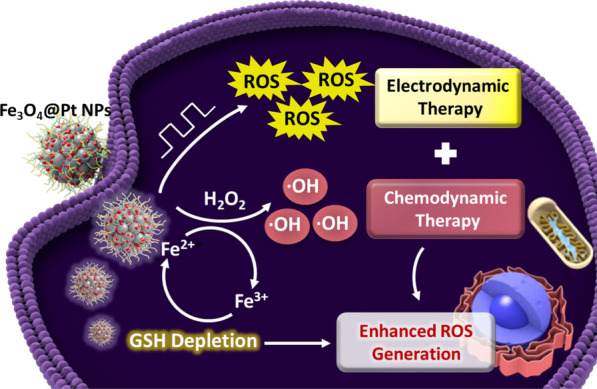

## Background

Reactive oxygen species (ROS), containing hydroxyl radical (·OH), superoxide (O_2_^·−^), singlet oxygen (^1^O_2_) and hydrogen peroxide (H_2_O_2_), widely exist in living organisms [1–3]. It plays a crucial role in physiological functions, which can modulate proteins, produce hormones, regulate cell signaling, mediate inflammation, and eliminate pathogens. However, excessive intracellular ROS induces the damage of proteins, lipids and DNA with no specificallity [4, 5], and thus cancer therapies based on ROS have been extenstively explored in the past years [6–10]. Recently, an emerged ROS-based cancer therapy, called as electrodynamic therapy (EDT) has attracted wide attention. The potential mechanism of EDT is to ultilize the catalytic reaction at the surface of platinum nanoparticles under an electric field to produce toxic ROS [11]. Several strategies have been explored to enhance the therapeutic efficacy of EDT. Porous platinum incorporated with GOx was designed and synthesized for oxygen-inductive starvation/EDT synergistic cancer therapy [12]. Porous platinum and its hybrids have been developed as drug delivery systems of anticancer drug for combinational chemotherapy and EDT [13, 14]. EDT offers some superiority comparing to current “dynamic” cancer therapies, including controllable ROS generation by electric field, independence to O_2_ and H_2_O_2_, and low side effect [13, 14]. Nonetheless, EDT also inherits the intrinsic shortcomings of ROS-based therapies, especially restrains from the complicated tumor microenvironment (TME).

Tumor microenvironment refers to the surrouding microenvironment where tumor cells are located and it play a influencial role to the occurrence, growth and metastasis of tumors [15, 16]. A groundswell of studies has been carried out to regulate diverse characteristics of TME such as acidity, hypoxia, overexpressed hydrogen peroxide (H_2_O_2_), and others. Glutathione (GSH), which is often upregulated in TME, serves as one of antioxidants in protecting cancer cells from oxidative damage by free radicals [17–23]. Therefore, the efficacy of ROS-based treatments is significantly hindered. Several strategies have been develpoed to reduce GSH content in TME. One strategy is to use a type of inhibitor of γ-glutamylcysteine synthetase, l-buthionine sulfoximine (BSO), to suppress the GSH production from the source [18, 24]. Another is to oxidize the GSH to glutathione disulfide (GSSG) by certain oxidizers [23, 25, 26]. In addition, glutathione peroxidase (GSH-Px) are able to catalyze the reduction of H_2_O_2_ with GSH as reductant [27]. In this process, GSH can be consumed and converted into GSSG by the oxidation of H_2_O_2_ [28], favoring current ROS-based therapies.

On the other hand, TME can also be ultilized as a portal for specific and effective treatment for tumors. Chemodynamic therapy (CDT), which exploits the TME features including acidity and overexpressed H_2_O_2_, is another ROS-based cancer therapy [29–31]. In the acidic TME with no external stimulus, the toxic ·OH can be produced in an in situ manner by catalyzing H_2_O_2_ via Fenton or Fenton-like reactions [32–35]. While CDT shows smart characteristics in utilizing the physiological conditions of TME, the lacking in the control of its ROS induction and thus therapeutic outcomes hinders its potential clinical translation. The armour of EDT with chemodynamic phenomenon and GSH depletion is therefore considered as a highly potential approach to amplify the advantages of two ROS-based methodologies by utilizing both internal and external stimulus. However, this ambitious integration in one therapeutic platform has yet been attempted, to the best of our knowledge.

**Fig.1:**
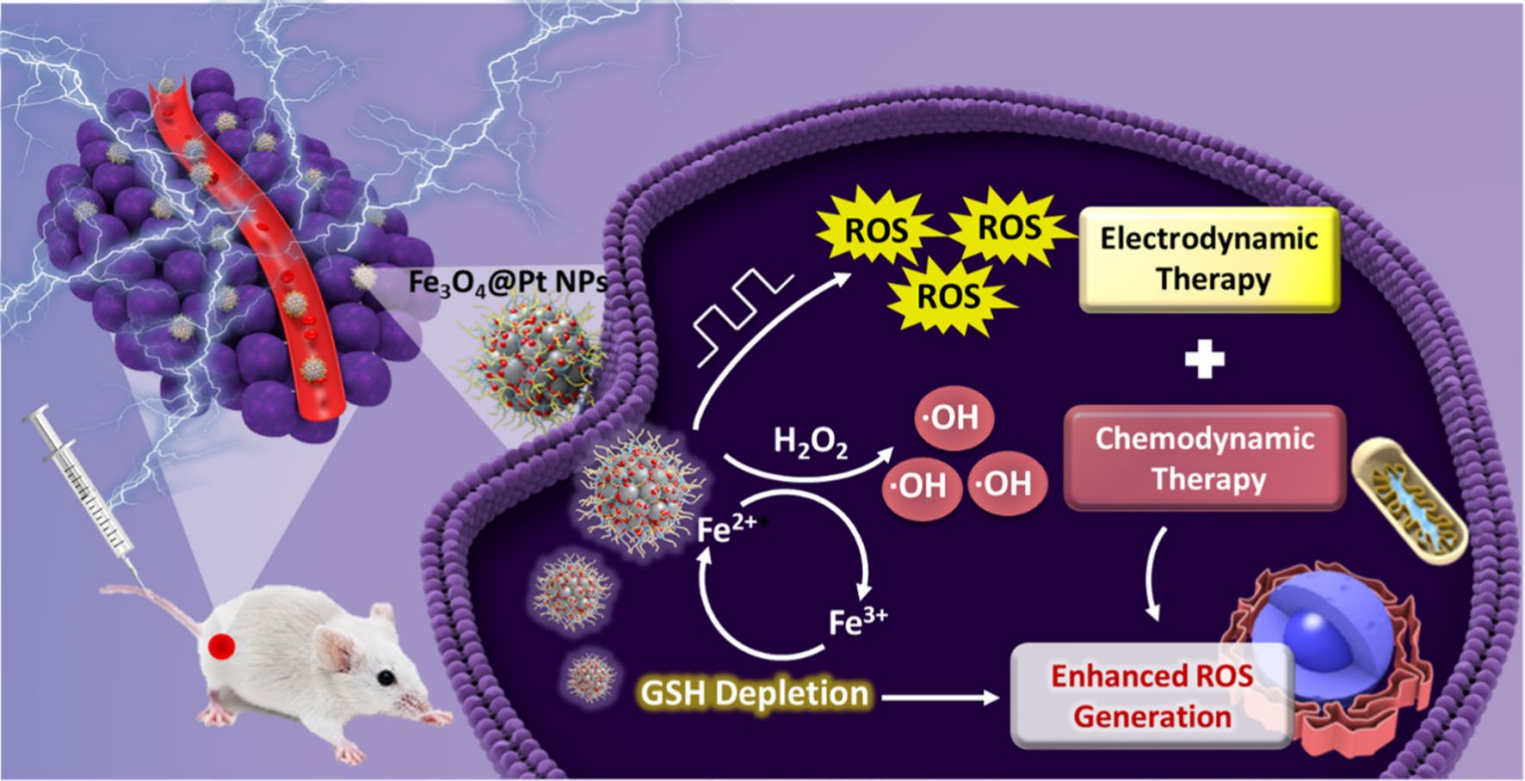
Schematic illustration of Fe_3_O_4_@Pt NPs for synergistic electrodynamic/chemodynamic tumor therapy with GSH depletion

Here for this purpose, iron oxide nanoparticles (Fe_3_O_4_ NPs) are synthesized and decorated with platinum nanocrystals at the surface (Fe_3_O_4_@Pt NPs). After reaching the tumor site as demonstrated in Fig. 1, the platinum nanoparticles (Pt NPs) disassembled from Fe_3_O_4_@Pt serve as an electro-sensitizer to generate ROS under an electric field. Meanwhile, Fe_3_O_4_ NPs release Fe^2+^ under the acidic condition and catalyze H_2_O_2_ to produce toxic ·OH through Fenton reaction. In addition, the released-Fe^3+^ deplete GSH effectively through redox reaction to inhibit potential ROS clearance, and generate more Fe^2+^ to facilitate the ·OH induction. In consequence, both in vitro and in vivo studies indicate Fe_3_O_4_@Pt NPs have enabled significant antitumor effect comparing to each of solo therapeutic modality. The study is therefore anticipated to offer a distinctive concept in designing multi-stimulus responsive systems for potential tumor treatments.

## Materials and methods

### Chemical and reagents

Ferric chloride hexahydrate (FeCl_3_·6H_2_O), ethylene glycol (EG), diethylene glycol (DEG), sodium acetate (CH_3_COONa, NaOAc), polyvinylpyrrolidone (PVP, K-30), sodium borohydride (NaBH_4_) and ethanol were all purchased from Sinopharm Chemical Reagent Co., Ltd. 3-aminopropyltriethoxysilane (APTES), polyacrylic acid (PAA, MW ≈ 1800, 50%), Amino group terminated poly (ethylene glycol) (PEG-NH_2_, 5 K) and potassium hexachloroplatinate (K_2_PtCl_6_) was purchased from Aladdin. All chemicals were not further purified before use.

### Synthesis of and modification of Fe_3_O_4_ NPs

According to a previous study [36], Fe_3_O_4_ NPs were prepared by solvent thermal method. 2 mmol FeCl_3_·6H_2_O was added into a mix solution of 6 mL ED and 14 mL DEG and the solution was stirred magnetically at room temperature for 30 min. Then, 2 g PVP was put into above mixture and the solutions were stirred magnetically at 125 ℃ for 1 h. After that, 1.5 g NaOAc was added. Another 0.5 h later, the solutions were poured into a 50 mL stainless-steel autoclave with Teflon lining and heated for 12 h at 200 ℃. Finally, Fe_3_O_4_ NPs were obtained by centrifugation and washed with ethanol and water for three times. To obtain amino modified Fe_3_O_4_ NPs, 10 mg Fe_3_O_4_ NPs were dispersed in 5 mL ethanol and then a stable solution was obtained by ultrasound. Next, 150 μL APTES was put into the solution. After stirring magnetically at 50 ℃ for 24 h, excess APTES was removed by magnetic separation and washed by ethanol.

### Synthesis and modification of Fe_3_O_4_@Pt NPs

The obtained 10 mg Fe_3_O_4_-NH_2_ NPs were put into 10 mL DI water and 25 mg K_2_PtCl_6_ was added. After ultrasonication for 1 h, 35 mg NaBH_4_ was put into 5 mL ice-cold water to obtain the solution and then it was added into above solution dropwisely. After ultrasonication for 1 h, the Fe_3_O_4_@Pt NPs were separated by magnet from the solution and washed several times by DI water. The obtained Fe_3_O_4_@Pt NPs were put into 10 mL DI water to get the solution by ultrasound and then 250 μL PAA solution was added. Next, NaOH was used to adjust the pH of solution to 8.0. After stirring overnight, the nanoparticles were separated by magnet and washed by DI water to remove excessive PAA. Subsequently, products were put into 8 mL DI water. 5 mg EDC and 10 mg mPEG-NH_2_ were added into solution, and purified by magnetic separation after 24 h. The final sample was washed by DI water for three times and collected for further use.

### Fenton reaction activity and Michaelis–Menten kinetics studies

The activity of Fenton reaction of Fe_3_O_4_ NPs was measured by 3,3ʹ,5,5ʹ-tetramethylbenzidine (TMB) via the chromogenic reaction (λ = 652 nm). Phosphate buffer solution (pH = 6) was prepared as the reaction buffer, and 100 mM H_2_O_2_ was added. The steady-state kinetic catalytic activities of 200 μg/mL Fe_3_O_4_ NPs were investigated by monitoring the absorbance variation at 652 nm upon addition of a series H_2_O_2_ concentration (10, 20, 50, 80, 100 mM). The rate of absorbance variation was used to calculate the change velocity of TMB concentration by the Beer–Lambert law (*A* = *εlc*, where *ε* = 39 000 M^−1^ cm^−1^, *l* = 10 mm). The H_2_O_2_ concentrations were used as an abscissa, and the rates of change in TMB concentration were used as an ordinate to build scatter diagram. It could be fitted by the Michaelis–Menten equation, known as Eq. (1)

1 $$y = V_{max} *x/\left( {K_{M} + x} \right)$$

where $\mathrm{y}$ is the velocity, ${V}_{max}$ is the maximal velocity, $x$ is the substrate concentration, and ${K}_{M}$ is the Michaelis–Menten constant.

Through a feasible deformation, the original Michaelis–Menten equation could be converted to Eq. (2)

2 $$\frac{1}{y} = \frac{{K_{M} }}{{V_{max} }}*\frac{1}{x} + \frac{1}{{V_{max} }}$$

### Electro-catalytic activity studies

The catalytic reaction activity driven by electric field was investigated by methylene blue (MB) via the degradation reaction. The equipments were similar to the previuous study [2]. Typically, MB (2.5 × 10^−5^ M) and Fe_3_O_4_@Pt NPs (200 μg/mL for Fe_3_O_4_) in 2 mL PBS was added into 24-well plate. The square wave AC electric field (10 mHz, 10 mA) was chosen following previous studies [2]. At different point-in-time, 50 μL of solution was sucked out and diluted to 200 μL by PBS. The absorption spectrum of MB were examined by UV–vis spectroscopy. Similarly, the MB degradation at different concentration (50 to 300 μg/mL) of Fe_3_O_4_@Pt NPs was studied.

### Depletion of GSH

The GSH depletion was examined by 5, 5ʹ-dithiobis-(2-nitrobenzoic acid) (DTNB). Fe_3_O_4_ NPs solutions (2 mg/mL) was added with GSH (1 mM) and put in the shaker at 37 ℃. At different points of time, 500 µL mL solution was centrifuged to remove Fe_3_O_4_ NPs. And 2.5 mL PBS, and 50 µL DTNB (10 mM) were added into the supernatant. The absorption spectrum was obtained by UV–vis spectroscopy.

### Releasing of Fe^2+^

The consumption of Fe^2+^ was examined by 1,10-phenanthroline. Fe_3_O_4_ NPs was incubated with or without GSH (10 mM) under buffer solution with different pH (7.4 or 6.0) in the shaker at 37 ℃. At different points of time, 1 mL of supernatant was collected by centrifugation and 1 mL of 1,10-phenanthroline (2 mg/mL) was added into the supernatant and the absorption spectrum was investigated.

### Cell viability assay

1 × 10^4^ 7702 cells were seeded into one well of 96-well plate and cultured for 12 h at 37 ℃. Consequently, different concentrations Fe_3_O_4_@Pt NPs were added into cells.

For CDT treatment, 4T1 cells were plated as above. The pH of medium was adjusted to 6 by HCl to simulate the acidic TME. Then, the medium (pH = 6) containing Fe_3_O_4_@Pt NPs at varied concentrations of 6, 12, 25, 50, 100, 200 μg/mL for Fe_3_O_4_ containing 100 μM H_2_O_2_.

For EDT treatment, 1 × 10^5^ 4T1 cells were seeded into one well of 24-well plates. After incubated with Fe_3_O_4_@Pt NPs at varied concentrations of 6, 12, 25, 50, 100, 200 μg/mL for Fe_3_O_4_ for 4 h, the cells were conducted by square wave AC electric field (10 mHz, 5 mA) for 10 min.

For combinational therapy, 4T1 cells were cultured with Fe_3_O_4_@Pt NPs at varied concentrations of 6, 12, 25, 50, 100, 200 μg/mL for Fe_3_O_4_ containing 100 μM H_2_O_2_ for 4 h, then conducted by square wave AC electric field (10 mHz, 5 mA) for 10 min.

For studying enhanced EDT of the GSH depletion by Fe^3+^, 4T1 cells were cultured with Pt NPs or Pt NPs plus Fe^3+^ at varied concentrations of 6, 12, 25, 50, 100, 200 μg/mL for Pt NPs for 4 h, conducted by square wave AC electric field (10 mHz, 5 mA) for 10 min.

All the cell viabilities were measured by CCK-8 assay by a microplate reader after another 24 h.

### Intracellular ROS generation

4T1 cells were treated with Fe_3_O_4_@Pt (200 μg/mL), Fe_3_O_4_@Pt plus H_2_O_2_ (100 μM), Pt plus electric field (5 mA, 10 min), Fe_3_O_4_@Pt plus electric field, Fe_3_O_4_@Pt plus H_2_O_2_ and electric field for 4 h. Then, before EDT treatment, cells were treated by DCFH-DA probe for 30 min. After another 1 h, the green fluorescence by DCFH was measured by the fluorescence microscopy.

### Intracellular GSH variation

4 × 10^5^ 4T1 cells were seeded into one well of 6-well plates. After treated with different samples for 12 h, the cells were centrifugated, washed three times by PBS and lysed by Triton-X-100 lysis buffer. The supernatants were collected by centrifugation and then 50 μL supernatants was sucked out and added with DTNB solution (50 μL, 400 μM). The GSH level was evaluated by absorption of DTNB at 412 nm using microplate reader after cultured for 30 min.

### In vivo study

All animal experiments were performed in accordance with the guidelines of the animal ethics committee of the Biological Resource Centre of the Agency for Science, Technology and Research, Zhejiang University. The Female BALB/c mice were bared with 4T1 tumors for in vivo study. The initial tumor volume was ~ 400 mm^3^. All the mice were divided to six groups: (1) PBS; (2) E; (3) Pt (4 mg/mL, 200 μL); (4) Fe_3_O_4_@Pt (4 mg/mL for Fe_3_O_4_, 200 μL); (5) Pt (4 mg/mL, 200 μL) + E; (6) Fe_3_O_4_@Pt (4 mg/mL for Fe_3_O_4_, 200 μL) + E. After intravenous injected with different samples for 24 h, group 2, 5 and 6 mices were conducted by the square wave AC field (10 mHz, 5 mA, 5 min). The tumor lengths, widths and the body weights were measured every 2 days. The tumor volumes were able to be calculated by the equation (volume = width^2^ × length /2).

The tumors from different groups were sucked out after 24 h. The tumors were sliced for H&E staining, fluorescent TUNEL staining and Ki67 staining and observed by a confocal microscopy.

### Statistical analysis

Data are expressed as mean ± standard deviation. Statistical significance was evaluated using the Student t-test to compare the results between two groups. P values < 0.05 were considered statistically significant (NS, no significance, *p < 0.05, **p < 0.01, ***p < 0.001).

## Results and discussion

### Synthesis of Fe_3_O_4_@Pt NPs

**Fig. 2:**
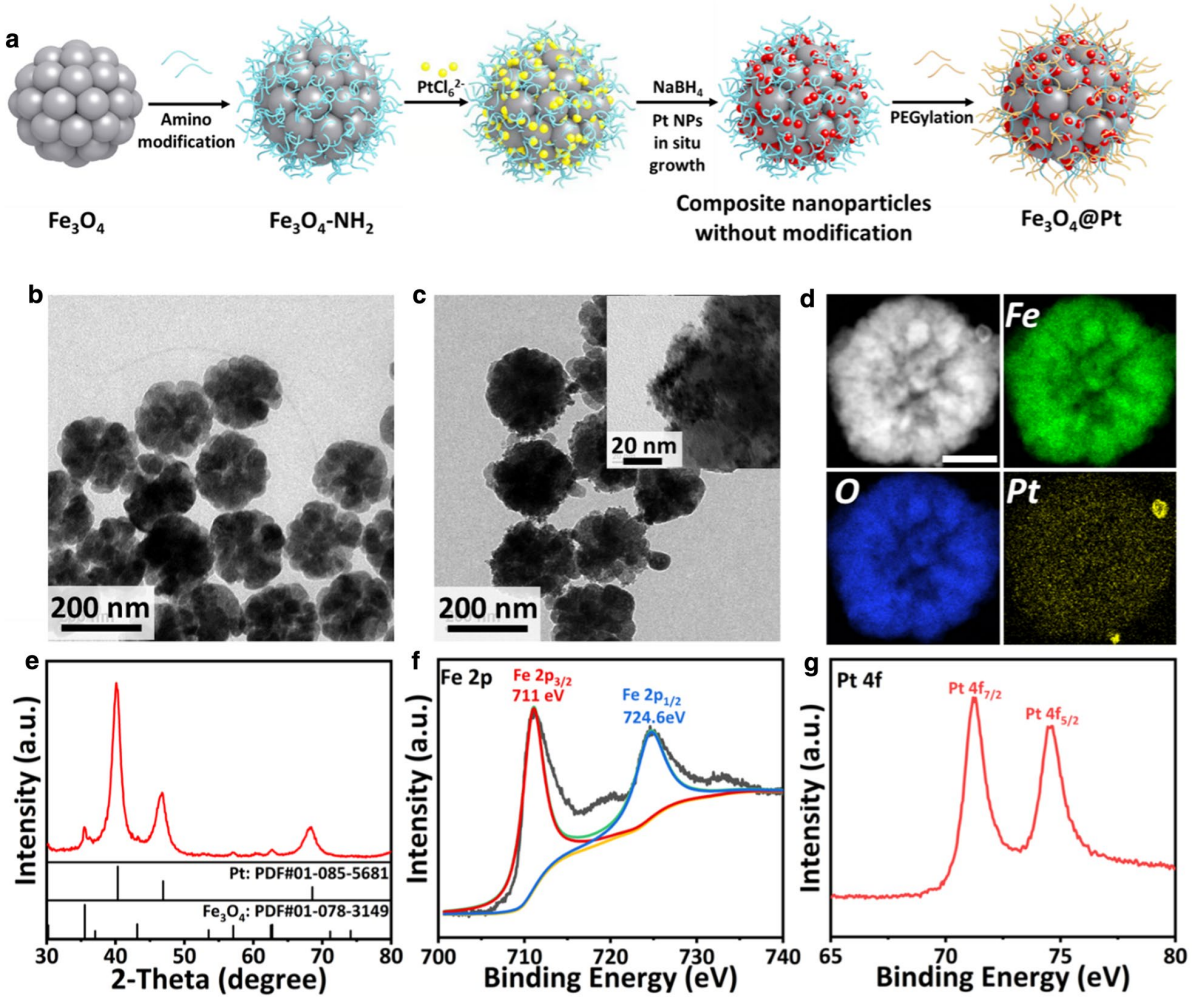
Synthesis and characterization of Fe_3_O_4_@Pt NPs. **a** Schematic illustration of synthesis procedure. TEM images of **b** as-prepared Fe_3_O_4_ NPs and **c** Fe_3_O_4_@Pt NPs. **d** Electron diffraction pattern and **e** XRD pattern of Fe_3_O_4_@Pt NPs. The surface valence status of **f** Fe and **g** Pt elements of Fe_3_O_4_@Pt NPs examined by the X-Ray photoelectron spectroscopy

Fe_3_O_4_ NPs were synthesized and modified with amino groups before the in situ growth of Pt NPs on the surface. Fe_3_O_4_@Pt NPs were modified with polyethylene glycol (PEG) to enhance its stability (Fig. 2a). The as-prepared Fe_3_O_4_ NPs are well dispersed and uniform with a size of ~ 200 nm (Fig. 2b and Additional file 1: Fig. S1). Presenting as clusters assembled by small iron oxide crystals, Fe_3_O_4_ NPs exhibit a rough surface morphology, favoring the subsequent growth and loading of Pt nanoparticles. The peaks in X-Ray photoelectron spectrum (XPS) of Fe_3_O_4_ are attributed to Fe 2p_3/2_ and Fe 2p_1/2_, as expected (Additional file 1: Fig. S2). Subsequently, K_2_PtCl_6_ was used as Pt sources and the NaBH_4_ was used as the reductant for the incorporation of Pt NPs. As shown in the TEM images, Pt nanocrystals are anchored at the surface of Fe_3_O_4_ NPs in a uniform manner (Fig. 2c). The elemental mapping of Fe_3_O_4_@Pt nanoparticles confirms that Pt element with high content presents across the nanoparticles in addition to Fe and O (Fig. 2d). The diffraction peaks exhibited in the XRD pattern are attributed to monoclinic Fe_3_O_4_ (PDF#01-078-3149) and cubic Pt (PDF#01-085-5681) (Fig. 2e). The doublet peaks in XPS of Fe_3_O_4_@Pt NPs match Fe 2p_3/2_, Fe 2p_1/2_ and Pt^0^ 4f_7/2_, Pt^0^ 4f_5/2_, respectively (Fig. 2f, g), demonstrating the co-existence of Fe_3_O_4_ and Pt in the nanocomposites. The hydrodynamic diameter of Fe_3_O_4_@Pt NPs is ~ 300 nm, as examined by dynamic light scattering (DLS) (Additional file 1: Fig. S3). The varied zeta potential of nanoparticles, during the synthesis and surface modification formation, indicates that Fe_3_O_4_@Pt NPs are successfully modified with PEG groups at its surface (Additional file 1: Fig. S4). As a result, Fe_3_O_4_@Pt NPs maintain stably dispersed in water, PBS and RPMI-1640 and FBS solution for 12 h, indicating its excellent solubility and stability for following in vitro and in vivo assessments (Additional file 1: Fig. S5a, b).

### Fenton activity and electrodynamic properties

**Fig. 3:**
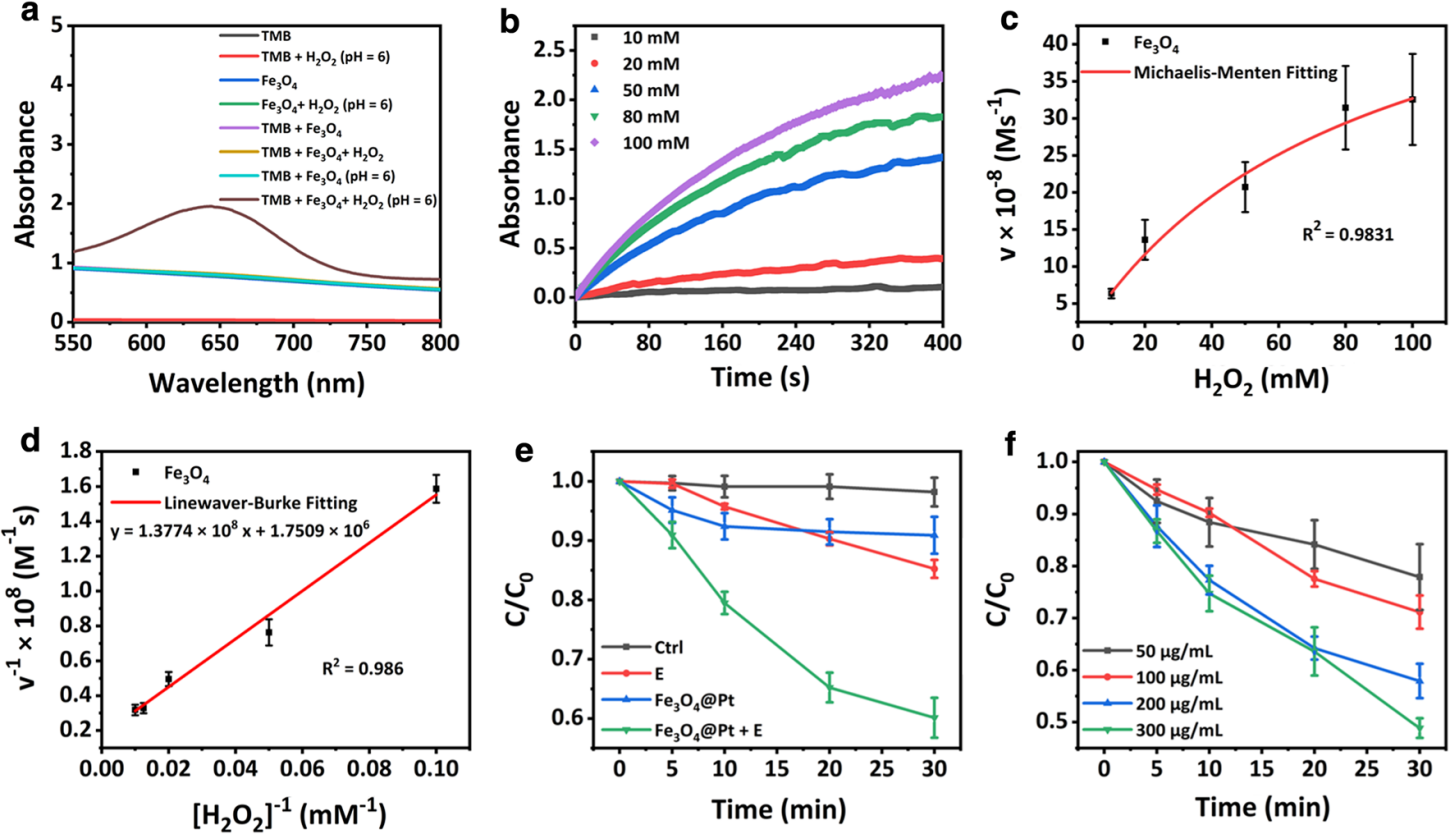
**a** UV–vis absorption spectra of the catalyzed oxidation of TMB (oxTMB) catalyzed by Fe_3_O_4_ NPs with 100 mM H_2_O_2_ under the reaction buffer (pH = 6). **b** Time-dependent absorbance changes at 652 nm as a result of the catalyzed oxidation of TMB at different H_2_O_2_ concentrations (10, 20, 50, 80, 100 × 10^−3^ M). **c** Michaelis–Menten kinetic analysis and **d** Lineweaver–Burk plotting for Fe_3_O_4_ with H_2_O_2_ as substrate. The steady-state catalytic rate (*v*) was calculated from the initial slopes of absorbance versus time plots in panel **b**. **e** Degradation rates of MB under different conditions ([Fe_3_O_4_@Pt]: 200 µg/mL for Fe_3_O_4_, AC output current: 10 mA, [MB]: 2.5 × 10^−5^ M). **f** Degradation rates of MB in the presence of Fe_3_O_4_@Pt NPs with different concentrations under electric field

In order to avoid the uncertainty in ROS evaluation induced by oxygen generated from Pt nanocrystals, the ·OH production of Fe_3_O_4_ NPs was examined to reveal the Fenton activity of Fe_3_O_4_@Pt NPs using 3,3′,5,5′-tetramethylbenzidine (TMB). As shown in Fig. 3a, in the TMB-H_2_O_2_ mixture solution with pH of 6, Fe_3_O_4_ NPs could effectively oxidate TMB to blue-colored TMB (oxTMB) which presents characteristic absorption peak at 652 nm, confirming the ·OH generation. To evaluate the catalytic activity, the catalytic kinetics was examined in a solution containing Fe_3_O_4_ NPs, TMB, and H_2_O_2_ at varied concentrations (10, 20, 50, 80, and 100 mM) in buffer solution (pH = 6). The findings show that higher H_2_O_2_ concentration induces more rapid increase of absorbance at 652 nm, implying accelerated ·OH generation (Fig. 3b).

Therefore, the inverse of H_2_O_2_ concentrations were used as an abscissa, and the inverse of the TMB concentrations-changing rates were used as an ordinate to make scatter diagram (Fig. 3c). The relationship could be fitted by Lineweaver–Burk plot, and ${K}_{M}$ and ${V}_{max}$ values are calculated to be 78.67 mM and 5.711 × 10^−7^ Ms^−1^, respectively (Fig. 2d).

The catalytic activity of Fe_3_O_4_@Pt NPs under square wave AC field was evaluated by the degradation assessment of MB under the neutral condition and without H_2_O_2_ [37]. The AC electric field was chosen to avoid the pH variation at the surrounding of electrodes. As shown in Fig. 3e and Additional file 1: Fig. S6a–d, the MB absorbance at 664 nm decreases effectively in the solution containing Fe_3_O_4_@Pt NPs (200 μg/mL) under electric field, while the absorption does not decrease in the control, Fe_3_O_4_@Pt NPs and AC electric field groups, as expected. The MB degradation triggered by Fe_3_O_4_@Pt NPs under electric field was further examined with varied particle concentrations (50, 100, 200, 300 μg/mL). The MB degradation is highly dependent to the particle concentration (Fig. 3f and Additional file 1: Fig. S7a–e). The velocity of MB degradation is significantly promoted as the increase of particle concentration. However, the addition of H_2_O_2_ does not induce clear influence on the MB degradation driven by Pt nanocrystals under electric field (Additional file 1: Fig. S8a–c).

### Synergistic promotion of ROS induction

**Fig. 4:**
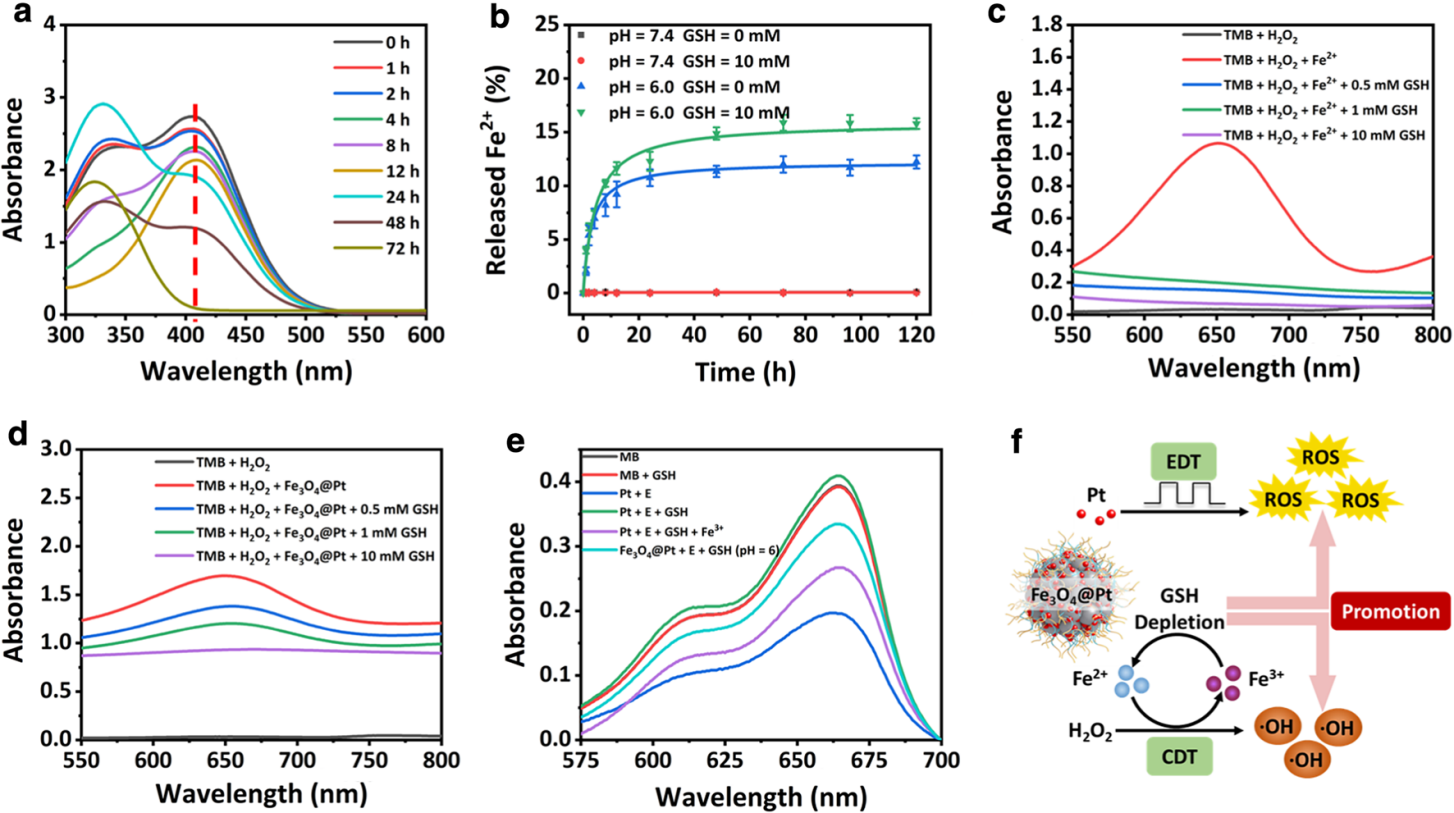
**a** UV–vis spectra of GSH solutions containing Fe_3_O_4_@Pt NPs with DTNB as the trapping agent of sulphydryl (-SH). **b** Release profiles of Fe^2+^ ions from Fe_3_O_4_@Pt NPs with 10 mM GSH. **c** UV–vis absorbance of TMB by Fe^2+^-mediated Fenton-like reaction in the presence or absence of GSH. **d** The UV–vis absorbance of TMB by Fe_3_O_4_@Pt NPs mediated Fenton-like reaction in the presence or absence of GSH. **e** MB degradation by Pt NPs and Fe_3_O_4_@Pt NPs-mediated EDT in the presence or absence of GSH and Fe^3+^. **f** Schematic illustration for the mechanism of ROS generation by Fe_3_O_4_@Pt NPs

It is known that the overexpressed GSH in tumor cells hinders the therapeutic efficacy of ROS-based cancer treatments. In this study, the functionality of Fe_3_O_4_@Pt NPs in the GSH depletion was explored. The nanoparticles were incubated in the solution containing excessive GSH, and the residual GSH was examined at different time points using a sulphydryl (-SH) indicator (DTNB). With the prolonged incubation time, the absorption peak at 412 nm decreases accordingly, demonstrating effective GSH depletion by the particles (Fig. 4a). To uncover the mechanism of GSH depletion by Fe_3_O_4_@Pt NPs, the releasing of Fe^2+^ ions from the core of Fe_3_O_4_@Pt NPs (Fe_3_O_4_) after incubation in GSH solution was examined by the UV–vis absorbance of 1,10-phenanthroline at 511 nm based on the standard curve of 1,10-phenanthroline solutions (Fig. 4b and Additional file 1: Fig. S9a, b). The release of Fe^2+^ can hardly be observed in the neutral environment. In contrast, more rapid release of Fe^2+^ is induced during incubation under an acidic condition (pH = 6), as expected. An accelerated increase in Fe^2+^ release was observed after the addition of 10 mM GSH, indicating that its reaction with GSH induces the effective transformation of Fe^3+^ to Fe^2+^. The TMB absorbance of ·OH induced by Fe^2+^ is significantly weakened in the presence of GSH, indicating that GSH eliminates the ·OH formed (Fig. 4c). In another words, the Fe_3_O_4_ core of Fe_3_O_4_@Pt NPs may effectively consume GSH, favoring the ·OH induction as well as the efficacy of CDT.

Meanwhile, the effect of Fe^3+^ mediated GSH depletion on the efficacy of EDT by Pt NPs was explored. As shown in Fig. 4e, Pt NPs effectively weaken the absorbance and degrade the MB solution under the electric field, while no clear degradation is induced in the presence of GSH (0.5 mM). However, after the addition of Fe^3+^, the MB degradation recovers effectively, indicating the effective comsumption of GSH by Fe^3+^. In addition, at the acid condition, Fe_3_O_4_@Pt NPs induce the recovery of MB degradation, indicating the it can deplete GSH by releasing Fe^3+^ in the acid environment. The findings suggest that Fe_3_O_4_@Pt NPs can enable enhanced EDT efficacy in the solution containing GSH.

In summary, the mechanism of the synergistic promotion in ROS induction achieved by Fe_3_O_4_@Pt NPs becomes clear (Fig. 4f). Despite the known facts of ROS induction from Pt-mediated EDT phenomenon and ·OH production from Fenton reaction by Fe_3_O_4_ core, the byproduct Fe^3+^ effectively consumes GSH contained in the solution. The GSH depletion achieved enables two crucial consequences. One is to supply extra Fe^2+^ due to the reaction of GSH consumption by Fe^3+^, favoring the ·OH production of CDT. Another is that the overall presence of ROS generated by both EDT and CDT phenomena is promoted, by a large magnitude, owing to the avoided ROS elimination by GSH. Therefore, Fe_3_O_4_@Pt NPs synthesized here have played multiple roles as excellent electrodynamic agent, Fenton agent for CDT and an effective GSH quencher, paving the way to its potential antitumor properties.

### In vitro anti-tumor properties

**Fig. 5:**
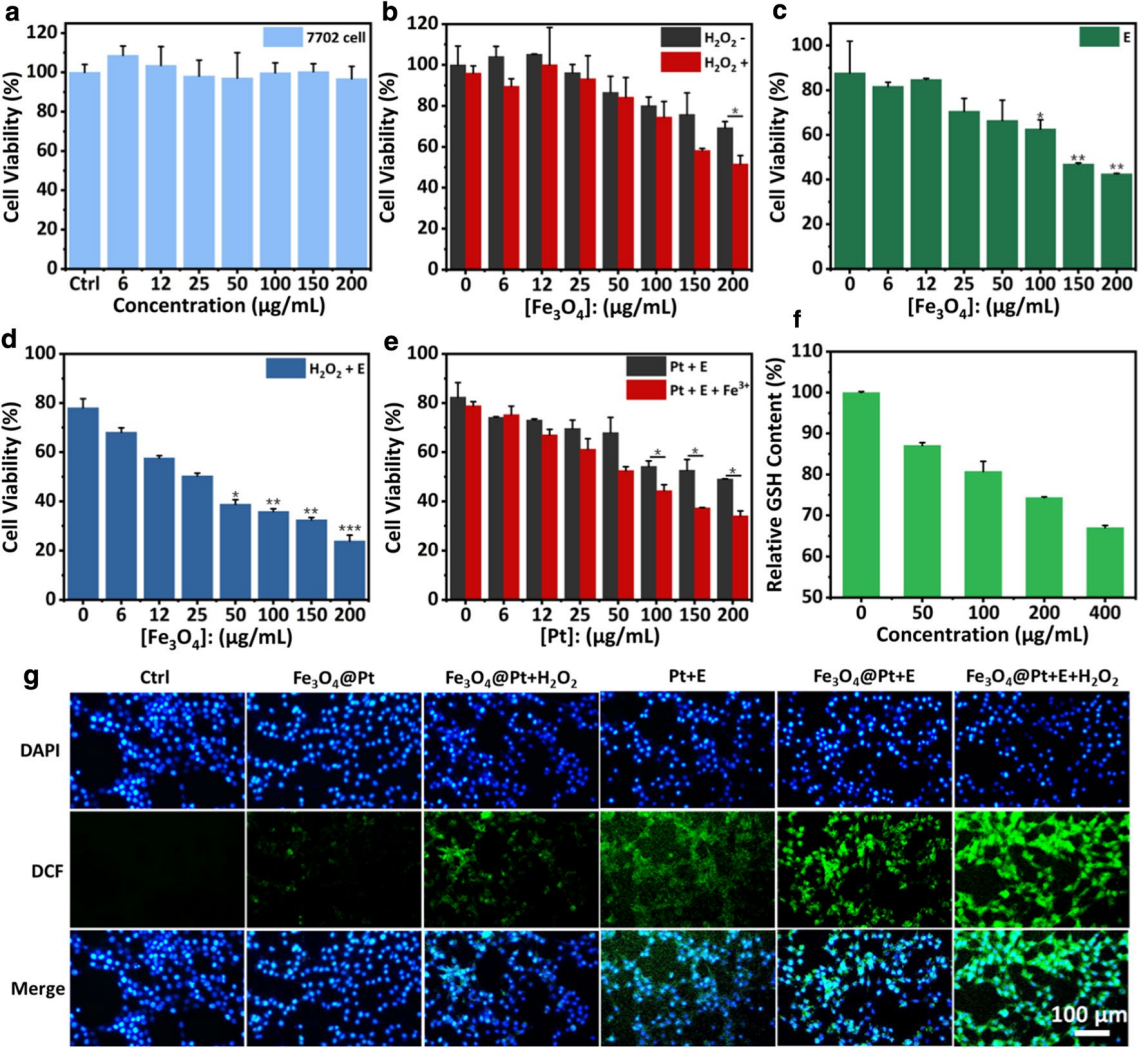
In vitro cell culture study. **a** Viabilities of 7702 cells with different concentrations of Fe_3_O_4_@Pt NPs. **b** Viabilities of 4T1 cells with different concentrations of Fe_3_O_4_@Pt NPs in the presence and absence of 100 μM H_2_O_2_. **c** Viability of 4T1 cells incubated with Fe_3_O_4_@Pt NPs under the electric field (5 mA, 10 min). **d** Viability of 4T1 cells incubated with Fe_3_O_4_@Pt NPs under the electric field (5 mA, 10 min) with 100 μM H_2_O_2_. **e** Viability of 4T1 cells incubated with Pt NPs at different concentrations under the electric field (5 mA, 10 min) in the presence and absence of Fe^3+^. **f** Relative intracellular GSH in 4T1 cells incubated with different concentration of Fe_3_O_4_@Pt NPs. **g** Intracellular ROS presence after different treatments using DCFH-DA as the ROS probe. (*p < 0.05, **p < 0.01 and ***p < 0.001 analyzed by Student’s t-test)

The cytotoxicity of Fe_3_O_4_@Pt nanoparticles was examined using 7702 cells by CCK-8. Fe_3_O_4_@Pt NPs at varied concentrations of 6, 12, 25, 50, 100, 200 μg/mL for Fe_3_O_4_ did not induce clear negative effect to cells after 24 h (Fig. 5a). In addition, 4T1 tumor cells were treated with Fe_3_O_4_@Pt NPs at varied concentrations of 6, 12, 25, 50, 100, 200 μg/mL for Fe_3_O_4_ with or without 100 μM H_2_O_2_ (pH = 6) for 24 h. It was observed that the cell killing efficacy was dependent on the concentration of Fe_3_O_4_@Pt NPs, as expected (Fig. 5b). When adding extra H_2_O_2_, an enhanced inhibition to cell variability occured due to the promoted ·OH production. Further, Fe_3_O_4_@Pt NPs at varied concentrations of 6, 12, 25, 50, 100, 200 μg/mL for Fe_3_O_4_ were incubated under the agitation by a square-wave electric field (5 mA) for 10 min. The cell variability was remarkably suppressed with increased particle concentration under the electric field owing to the enhanced EDT effect (Fig. 5c). The combined effect of CDT and EDT triggered by Fe_3_O_4_@Pt was further explored. As shown in Fig. 5d, Fe_3_O_4_@Pt NPs (200 μg/mL for Fe_3_O_4_) with 100 μM H_2_O_2_ (pH = 6) under AC electric field (5 mA, 10 min) induced distinctive inhibition to cell variability, higher than that from Fe_3_O_4_@Pt NPs under electric field or Fe_3_O_4_@Pt NPs with 100 μM H_2_O_2_ (pH = 6) but no electric field, indicating the enhanced in vitro antitumor effect of the combined CDT and EDT.

To reveal the favoring effect of CDT agent (Fe_3_O_4_) to the EDT efficacy induced by Pt nanocrystals, Pt NPs were incubated with tumor cells in absence and presence of Fe^3+^ and agitated by AC electric field for 10 min. It is clear that the addition of Fe^3+^ promoted the inhibition effect to cell variability under the AC electric field (Fig. 5e). The findings indicate that the intracellular GSH can be effectively consumed by Fe^3+^ and this may promote ROS presence induced by electrodynamic phenomenon of Pt NPs. The intracellular GSH level after being incubated with Fe_3_O_4_@Pt NPs at varied concentrations of 50, 100, 200, 400 μg/mL for Fe_3_O_4_ was quantitatively measured by the method of Ellman’s reagent (Fig. 5f). As expected, GSH content within cells decreased with the increased concentration of Fe_3_O_4_@Pt NPs, indicating the effective GSH consumption by the particles. In addition, the intracellular GSH levels after different treatments were investigated (Additional file 1: Fig. S10). The content of GSH in tumor cells treated with Fe_3_O_4_@Pt with electric field presented in the lowest magnitude comparing to other groups.

To uncover the in vitro antitumor mechanism of Fe_3_O_4_@Pt NPs, the intracellular ROS levels were examined using DCFH-DA probe, which show green fluorescence in the presence of ROS. Single treatments, including Fe_3_O_4_@Pt NPs, Fe_3_O_4_@Pt NPs plus H_2_O_2_, and Pt NPs under electric field, induced certain fluorescence. Notably, cells treated with Fe_3_O_4_@Pt NPs under electric field presented conspicuous green fluorescence, indicating considerably promoted ROS content. The supply of extra H_2_O_2_ during cell incubation may further improve intracellular ROS due to the enhanced Fenton reactions, as expected (Fig. 5g). Overall, Fe_3_O_4_@Pt NPs, presenting multiple tailored properties, can effectively enable promoted synergistic antitumor effect of CDT and EDT with GSH depletion.

### In vivo study

**Fig. 6:**
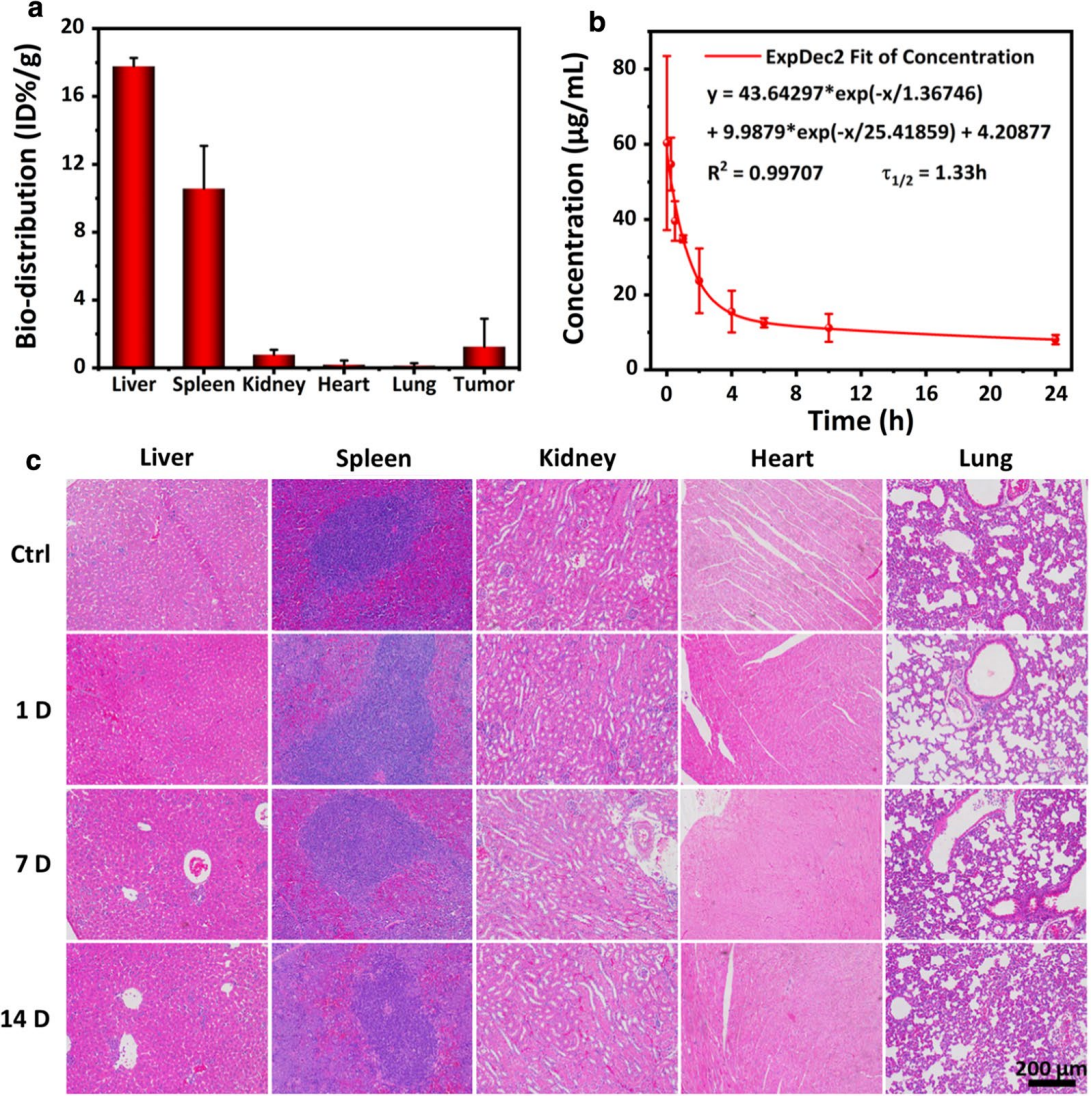
In vivo biodistribution and biosafety of Fe_3_O_4_@Pt NPs. **a** The biodistribution of Pt (% injected dose (ID) of Pt per gram of tissues) in main organs and tumor after intravenous administrations of Fe_3_O_4_@Pt NPs for 24 h; **b** The blood circulation curve of intravenously injected Fe_3_O_4_@Pt NPs. The half-time (τ_1/2_) is calculated to be ~ 1.33 h. **c** H&E staining of mice major organs (liver, spleen, kidney, heart and lung), to examine the histological variations after intravenous injection at 1, 7, and 14 days

To evaluate the biodistribution and biosafety, mice bearing 4T1 tumors were intravenously (i.v.) injected with Fe_3_O_4_@Pt NPs. The major organs were harvested after 24 h. The biodistribution study was carried out by examining the Pt content using ICP-OES (Fig. 6a). The accumulated content of Pt in the tumors reached ~ 1.25% of the injection dose. This Pt enrichment at the tumor site indicates the effective tumor accumulation of Fe_3_O_4_@Pt NPs after intravenous injection attributing to enhanced permeability and retention (EPR) effect. It is noteworthy that the accumulated content of Pt at the tumor site (~ 1.25%) is of consierably high magnitude for intravenous administration, as a variety of current nanoparticles reported reach by as low as ~ 0.7% [38]. In addition, in the blood-circulation experiment, a circulating half-life of ~ 1.33 h in blood stream was observed (Fig. 6b). The in vivo toxicity potential of Fe_3_O_4_@Pt NPs was assessed by histopathological analysis (Fig. 6c), and no apparent variation was observed in histopathological staining images of heart, lung, liver, spleen or kidney in 1, 7 and 14 days of the experimental period, comparing to the control group. Overall, the findings indicate that the synthesized Fe_3_O_4_@Pt NPs do not present apparent in vivo toxicity.

**Fig. 7:**
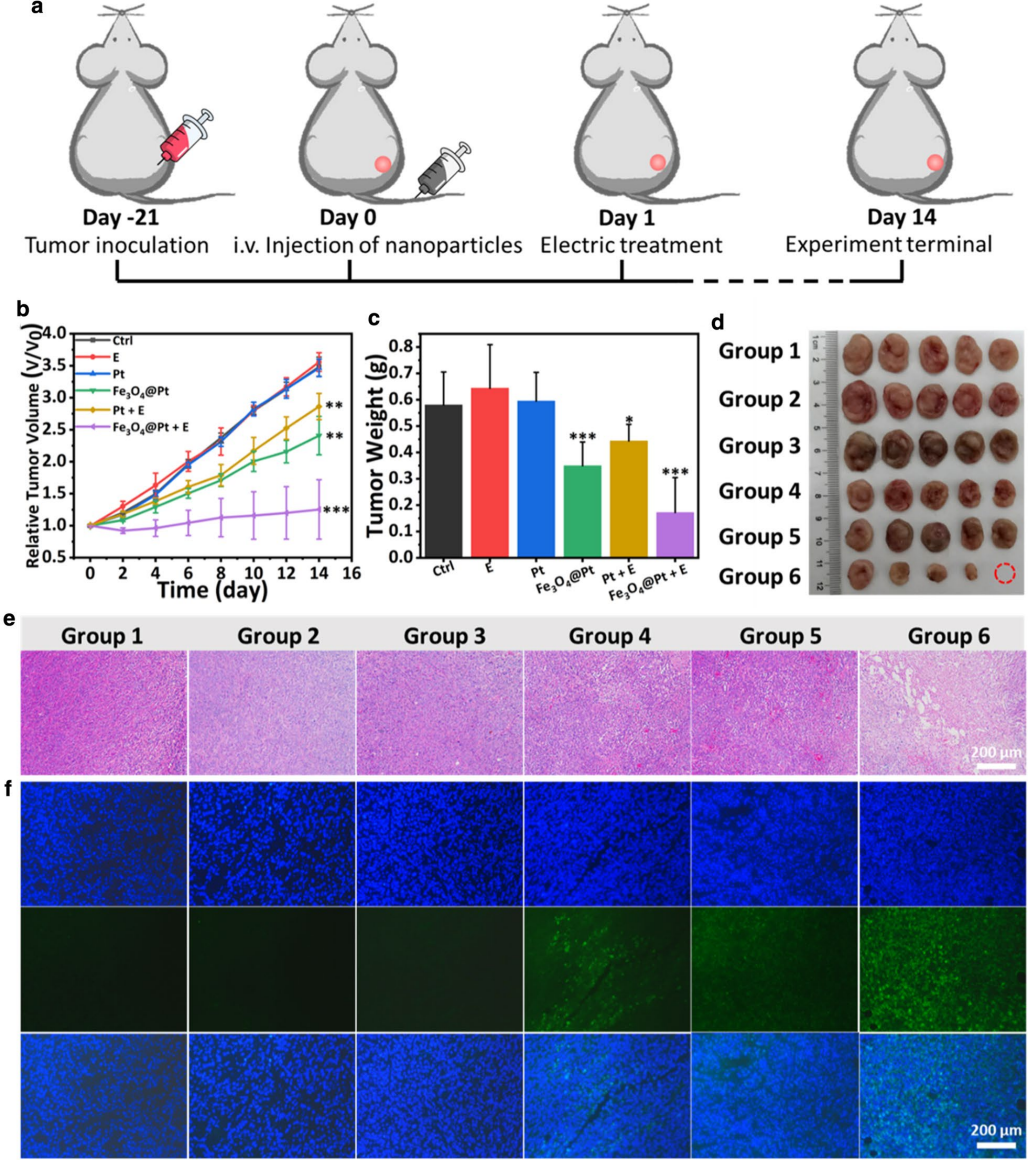
In vivo antitumor properties. **a** Schematic illustration of treatment procedures by Fe_3_O_4_@Pt in 4T1 tumor model. **b** Relative tumor volumes in mice after different treatment. **c** The average tumor weights and **d** digital photographs of tumors collected at day 14 from different groups of mice. **e** Microscopic images of H&E stained tumor slices after different treatments. **f** Microscopic images of immunofluorescence TUNEL-stained tumor slices. (*p < 0.05, **p < 0.01 and ***p < 0.001 analyzed by Student’s t-test)

Subsequently, the in vivo antitumor properties of the particles were revealed. The treatment protocol of animal experiments is illustrated in Fig. 7a. The experimental groups included the control (PBS) (Group 1), E (Group 2), Pt NPs (Group 3), Fe_3_O_4_@Pt NPs (Group 4), Pt NPs injection + E (Group 5) and Fe_3_O_4_@Pt NPs + E (Group 6). 200 μL Fe_3_O_4_@Pt NPs solution (4 mg/mL for Fe_3_O_4_), and E was maintained as the square-wave AC (5 mA, 10 mHz, 5 min).

The tumor length and width were recorded every 2 days by digital capliper. According to the equation, tumor volume was calculated and normalized to its initial size. As revealed in Fig. 7b, Group 2 and 3 showed negligible suppression to tumors, while Group 4 and 5 presented partially suppressed tumor growth. In addition, the tumor growth in Group 6 combining EDT and CDT exhibited the most significant tumor suppression in all the groups. The mice body weight were measured every two days (Additional file 1: Fig. S11). There were no apparent changes in body weight of mice, indicating negligible system toxicity of nanoparticles. After 14 days, tumors of all groups were collected, weighted and imaged (Fig. 7c, d). The tumor weight and volume from Group 6 were remarkablely inferior to that of other groups.

As shown in the H&E images, Group 6 exhibited the more dramatic cellular damage than the other groups (Fig. 7e). As shown in TUNEL fluorescence images, the samples of Group 1, 2 and 3 showed no clear apoptotic signals. Group 4 and 5 showed weak green signals. In contrast, Group 6 induced the significant apoptotic signals (Fig. 7f).

## Conclusions

In this study, we have designed and constructed a combinational EDT and CDT platform based on Fe_3_O_4_@Pt NPs for the first time. The particles do not only catalyze H_2_O_2_ to generate ·OH at acidic TME by Fenton reaction, but also show intrinsic ROS generation properties based on the catalytic reaction of Pt NPs triggered by the electric field. In particular, Fe_3_O_4_@Pt NPs can effectively deplete intracellular GSH by releasing Fe^3+^ in acidic TME, inhibiting the intrinsic ROS elimination of tumor cells. Both in vitro and in vivo studies show significant antitumor phenomena owing to the combinational effects of CDT, EDT and GSH depletion. This new therapeutic system appears to be highly effective in treating tumors with relatively large sizes (~ 400 mm^3^ at the initial state). This study has therefore offered a potential concept with distinctive functioning mechanism, and inspired future explorations in developing synergistic tumor therapeutic approaches with high efficacy.

## Supplementary Information

**Additional file 1: Figure S1.** Scanning electron microscopy image of as-prepared Fe_3_O_4_ nanoparticles. **Figure S2.** X-Ray photoelectron spectrum of Fe_3_O_4_ nanoparticles. **Figure S3.** Size distribution of Fe_3_O_4_@Pt NPs. **Figure S4.** Zeta potentials of Fe_3_O_4_@Pt NPs during the synthesis and surface modification by PEG. **Figure S5.** (a) Optical photographs of Fe_3_O_4_@Pt nanoparticles dispersed in water, phosphate buffered saline (PBS), RPMI-1640 cell culture and fetal bovine serum (FBS) for 12 h. (b) Size distribution of Fe_3_O_4_@Pt nanoparticles dispersed in water, PBS and RPMI-1640. **Figure S6.** UV–vis absorption spectra of MB solutions degraded under different conditions ([Fe_3_O_4_]: 200 µg/mL, AC output current: 10 mA,10 mHz, [MB]: 2.5 × 10^−5^ M). **Figure S7.** UV–vis absorption spectra of MB solutions degraded by Fe_3_O_4_@Pt with different concentrations (AC output current: 10 mA,10 mHz, [MB]: 2.5 × 10^−5^ M). **Figure S8.** (a) UV–vis absorption spectra of MB solutions degraded by Pt NPs under the 10 mHz AC field in the presence and absence of H_2_O_2_([Pt]: 200 µg/mL, output current: 10 mA, [MB]: 2.5 × 10^−5^ M, [H_2_O_2_]: 100 µM). (c) Degradation rates of MB in the presence of Pt NPs with or without H_2_O_2_. **Figure S9.** (a) UV–vis absorbance spectra of 1,10-phenanthroline solutions with different Fe^2+^ concentrations, and (b) the relationship between the optical absorbance at 511 nm and the concentration of 1,10-phenanthroline solutions. **Figure S10.** Relative intracellular GSH in 4T1 cells with different treatments. ([Fe_3_O_4_]: 200 µg/mL; electric field: square wave AC field; output current: 5 mA, time: 10 min). **Figure S11.** Average body weights of mice after different treatments.

## Data Availability

All data generated or analyzed during this study are included in this published article and its additional information files.
