# Supplementary material for: Fe3O4@Pt nanoparticles to enable combinational electrodynamic/chemodynamic therapy
Source: J Nanobiotechnology. 2021 Jul 10;19:206. doi: 10.1186/s12951-021-00957-7 (PMC8272323; doi:10.1186/s12951-021-00957-7)
Supplement: Supplementary file 1 — Additional file 1: Figure S1. Scanning electron microscopy image of as-prepared Fe3O4 nanoparticles. Figure S2. X-Ray photoelectron spectrum of Fe3O4 nanoparticles. Figure S3. Size distribution of Fe3O4@Pt NPs. Figure S4. Zeta potentials of Fe3O4@Pt NPs during the synthesis and surface modification by PEG. Figure S5. (a) Optical photographs of Fe3O4@Pt nanoparticles dispersed in water, phosphate buffered saline (PBS), RPMI-1640 cell culture and fetal bovine serum (FBS) for 12 h. (b) Size distribution of Fe3O4@Pt nanoparticles dispersed in water, PBS and RPMI-1640. Figure S6. UV–vis absorption spectra of MB solutions degraded under different conditions ([Fe3O4]: 200 µg/mL, AC output current: 10 mA,10 mHz, [MB]: 2.5 × 10^−5^ M). Figure S7. UV–vis absorption spectra of MB solutions degraded by Fe3O4@Pt with different concentrations (AC output current: 10 mA,10 mHz, [MB]: 2.5 × 10^−5^ M). Figure S8. (a) UV–vis absorption spectra of MB solutions degraded by Pt NPs under the 10 mHz AC field in the presence and absence of H2O2([Pt]: 200 µg/mL, output current: 10 mA, [MB]: 2.5 × 10^−5^ M, [H2O2]: 100 µM). (c) Degradation rates of MB in the presence of Pt NPs with or without H2O2. Figure S9. (a) UV–vis absorbance spectra of 1,10-phenanthroline solutions with different Fe2+ concentrations, and (b) the relationship between the optical absorbance at 511 nm and the concentration of 1,10-phenanthroline solutions. Figure S10. Relative intracellular GSH in 4T1 cells with different treatments. ([Fe3O4]: 200 µg/mL; electric field: square wave AC field; output current: 5 mA, time: 10 min). Figure S11. Average body weights of mice after different treatments. [file 12951_2021_957_MOESM1_ESM.docx]

**Fe_3_O_4_@Pt Nanoparticles to Enable Combinational Electrodynamic / Chemodynamic Therapy**

Tong Chen^1, ‡^, Qiang Chu^1, ‡^, Mengyang Li^1^, Gaorong Han^1^, and Xiang Li^1, 2 *^

^1^ State Key Laboratory of Silicon Materials, School of Materials Science and Engineering, Zhejiang University, Hangzhou, Zhejiang 310027, China

^2^ ZJU-Hangzhou Global Scientific and Technological Innovation Centre, Zhejiang University, Hangzhou, 311200, P.R. China

^‡^ Authors with equal contribution

*** Corresponding Author**

Xiang Li; E-mail: [xiang.li@zju.edu.cn](mailto:xiang.li@zju.edu.cn)

**Characterizations**

Scanning electron microscopy (SEM) images were taken by a scanning electron microscope (SU-70, Hitachi, Japan) and transmission electron microscopy (TEM) images were taken by a transmission electron microscope (HT7700, HITACHI, Japan). The elemental mapping was obtained by a JEOL electron microscope (JEM-2100, JEOL, Japan). The phase identification was investigated by X-ray diffraction instrument (XRD, X’ pert PRO MPD, Netherlands) with Cu Kα radiation (λ = 0.154 nm) and operating at 40kV and 40mA. The examination of X-ray photoelectron spectroscopy (XPS) was carried out using VG ESCALAB MKII spectrometer. The UV-Vis spectra were recorded by a spectrophotometer (UV2600, Shimadzu, Japan). The dynamic light scattering (DLS) zeta potential and size distribution were measured on a Malvern zetasizer (nano-ZS90, UK).


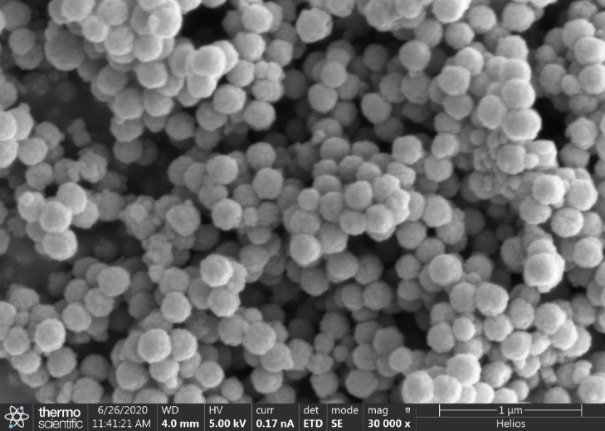


**Supplementary Figure 1.** Scanning electron microscopy image of as-prepared Fe_3_O_4_ nanoparticles.


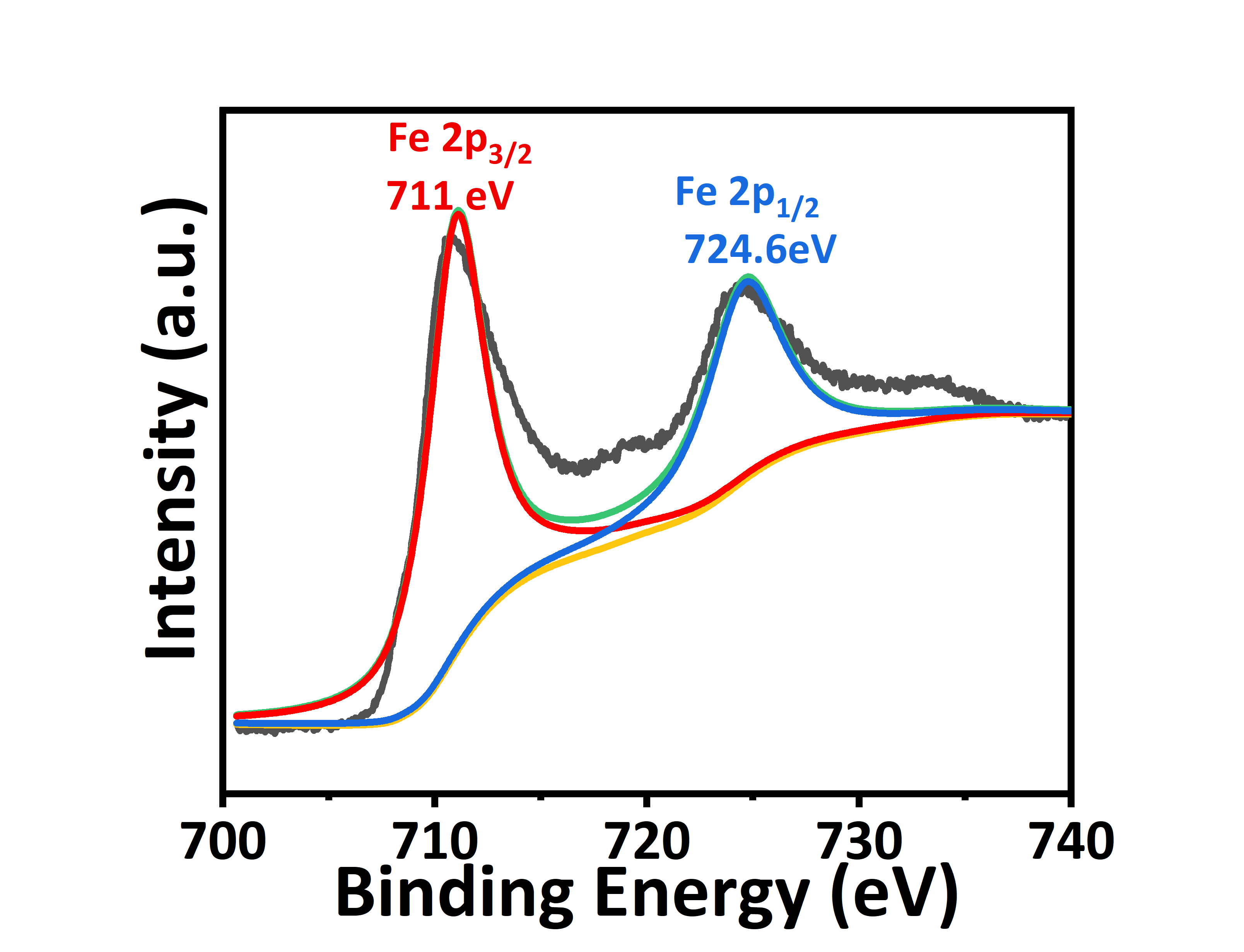


**Supplementary Figure 2.** X-Ray photoelectron spectrum of Fe_3_O_4_ nanoparticles.


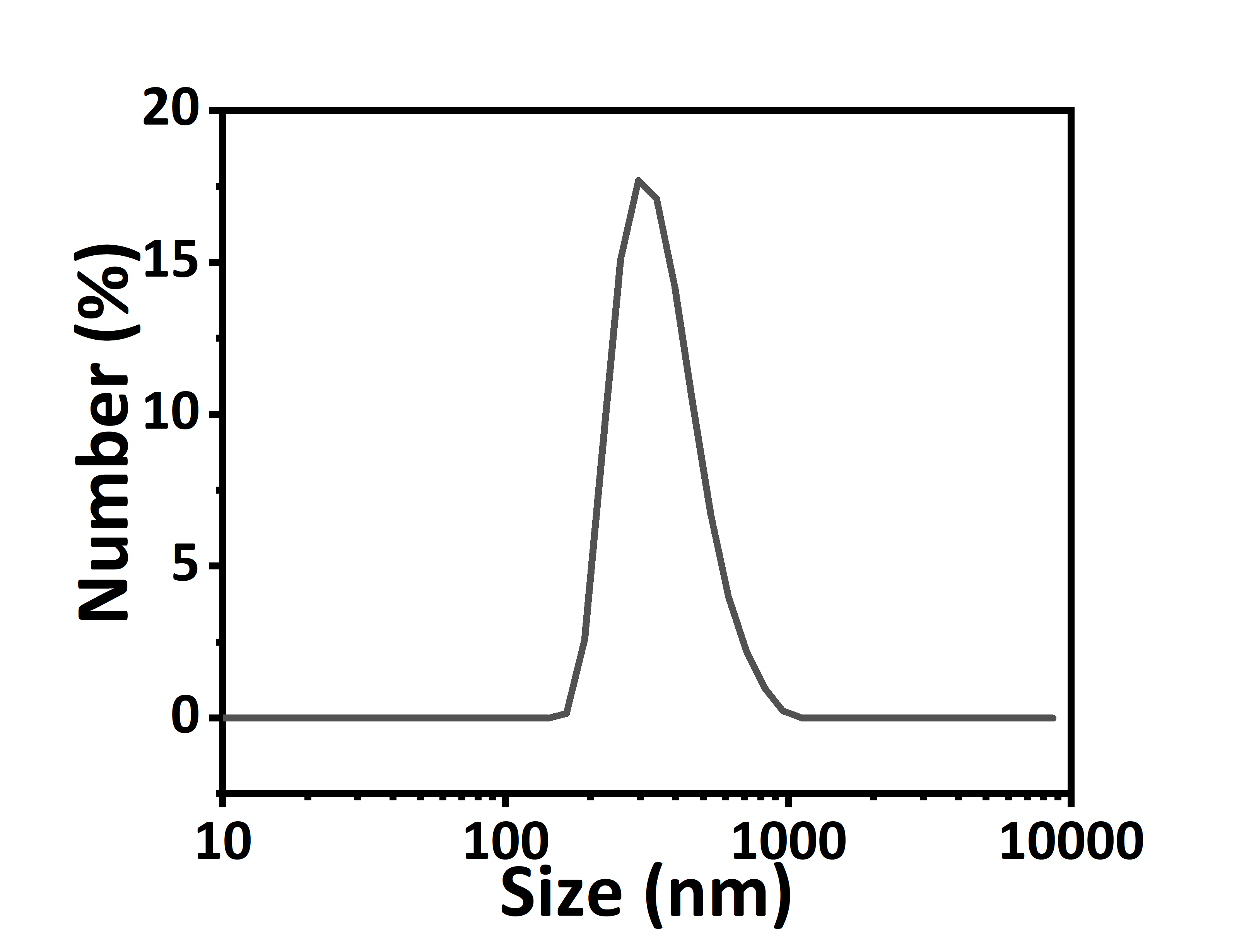


**Supplementary Figure 3.** Size distribution of Fe_3_O_4_@Pt NPs.


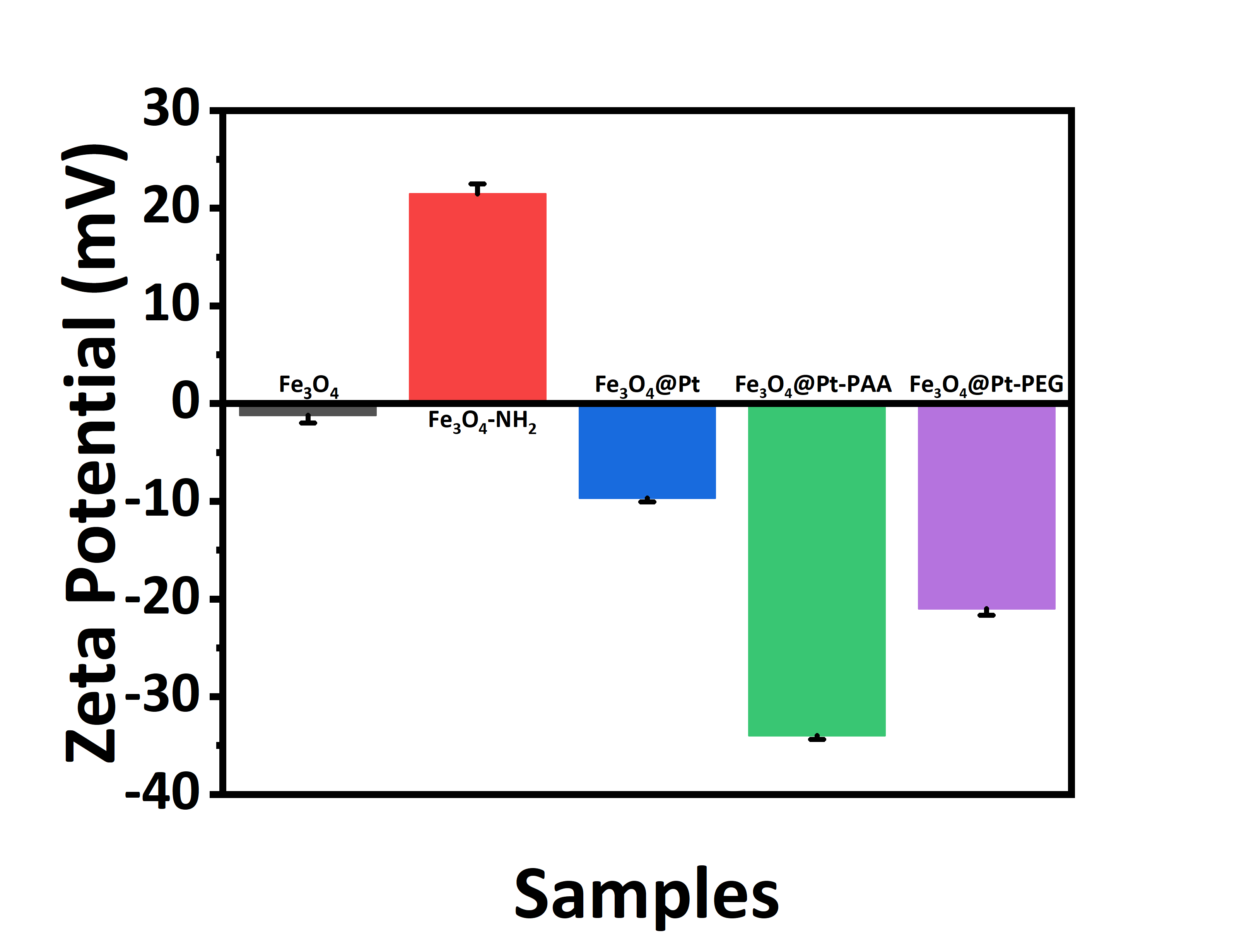


**Supplementary Figure 4.** Zeta potentials of Fe_3_O_4_@Pt NPs during the synthesis and surface modification by PEG.


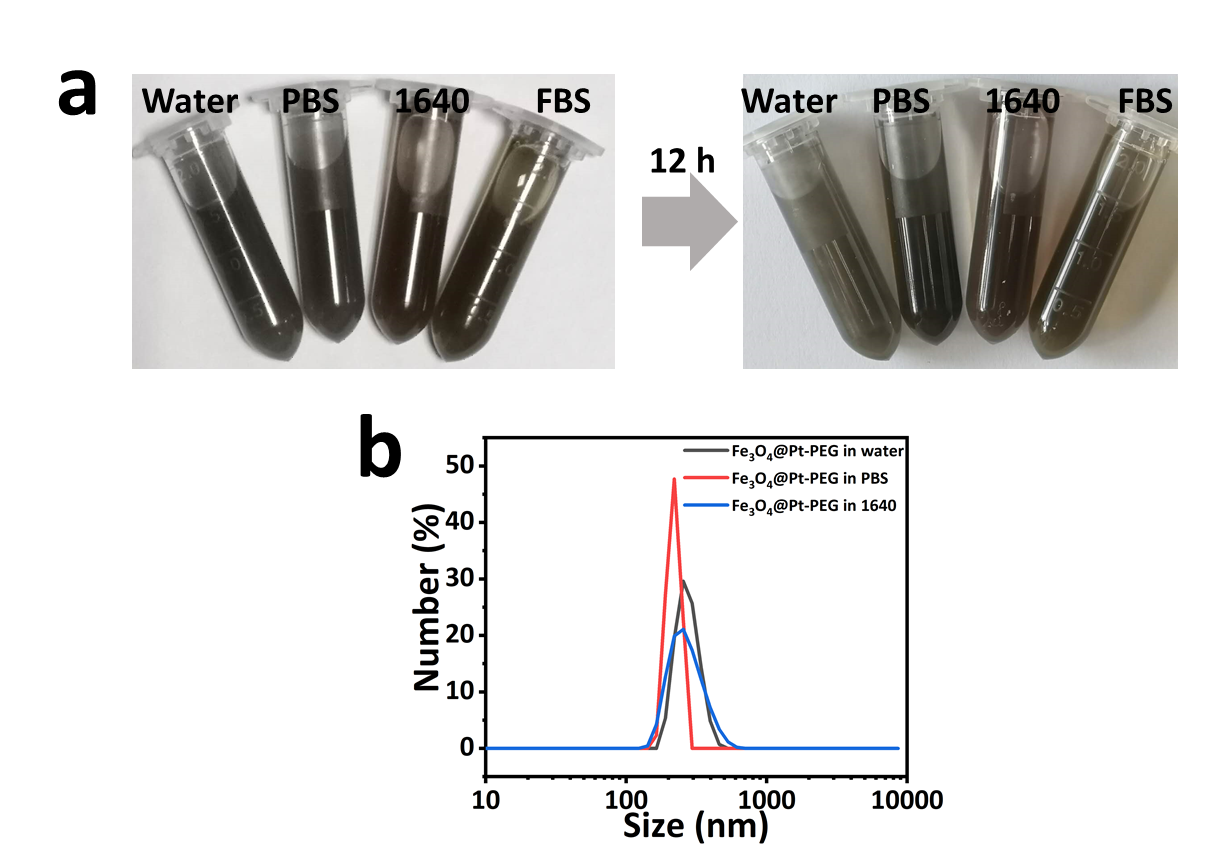


**Supplementary Figure 5.** (a) Optical photographs of Fe_3_O_4_@Pt nanoparticles dispersed in water, phosphate buffered saline (PBS), RPMI-1640 cell culture and fetal bovine serum (FBS) for 12 hours. (b) Size distribution of Fe_3_O_4_@Pt nanoparticles dispersed in water, PBS and RPMI-1640.


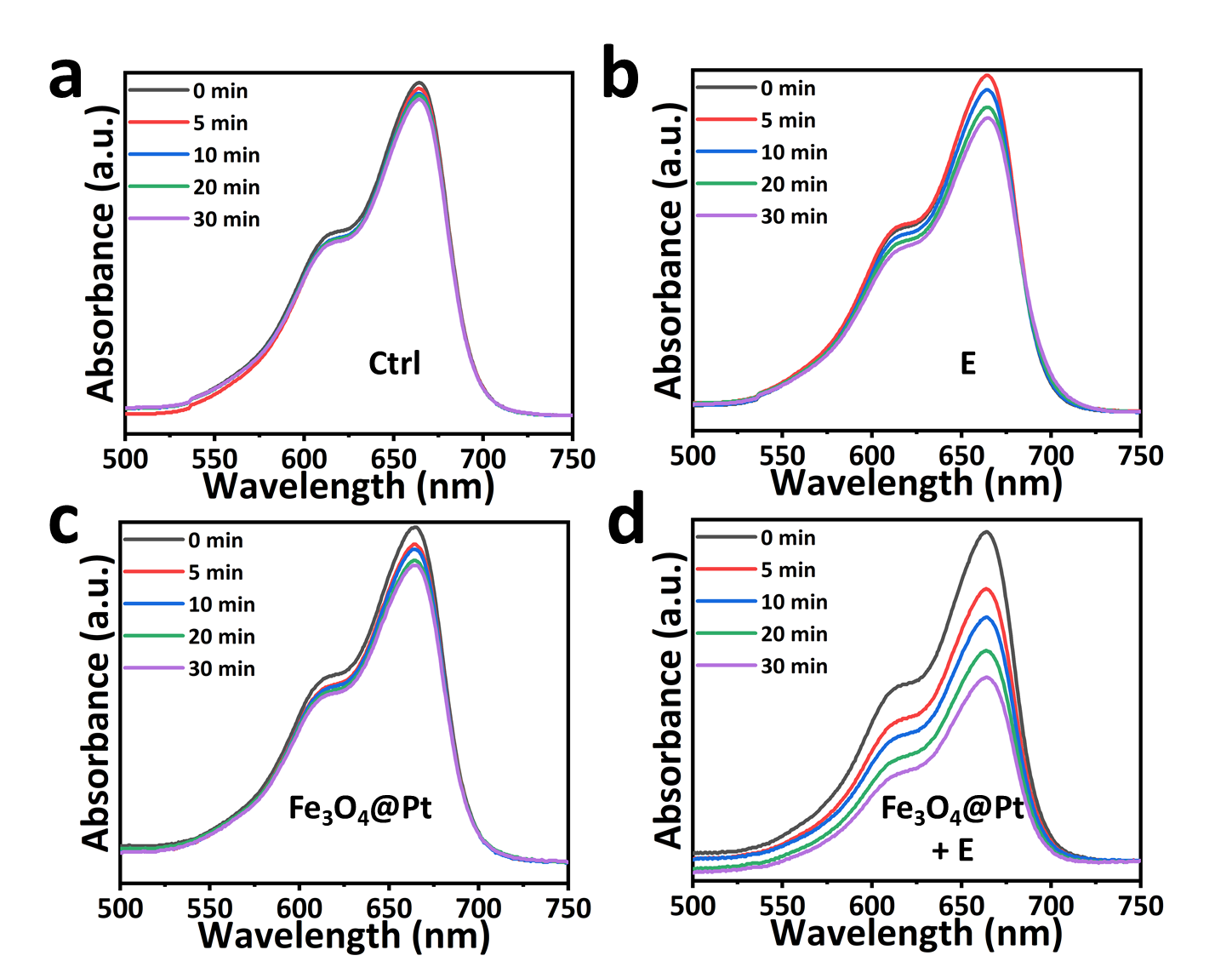


**Supplementary Figure 6.** UV-vis absorption spectra of MB solutions degraded under different conditions ([Fe_3_O_4_]: 200 µg mL^−1^, AC output current: 10 mA,10 mHz, [MB]: 2.5 × 10^−5^ M).


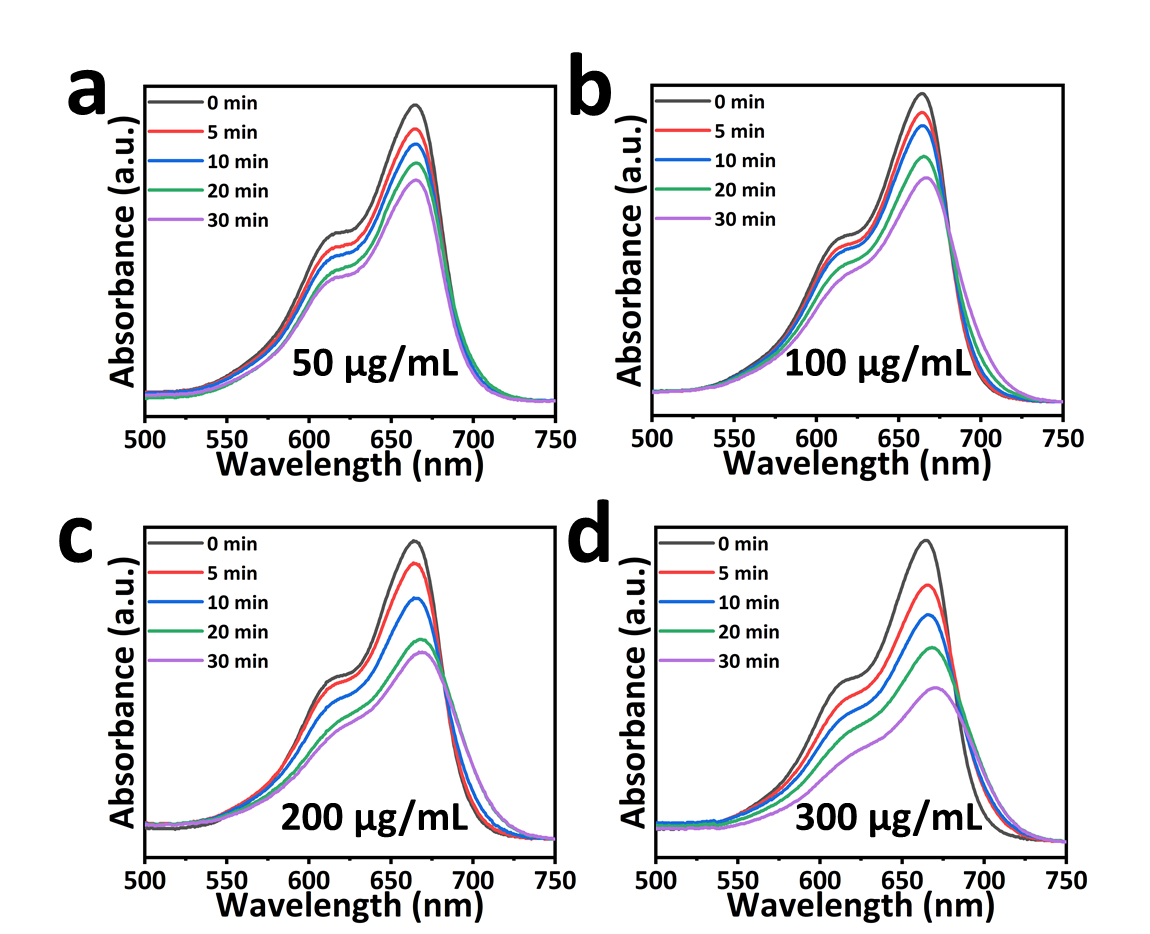


**Supplementary Figure 7.** UV-vis absorption spectra of MB solutions degraded by Fe_3_O_4_@Pt with different concentrations (AC output current: 10 mA,10 mHz, [MB]: 2.5 × 10^−5^ M).


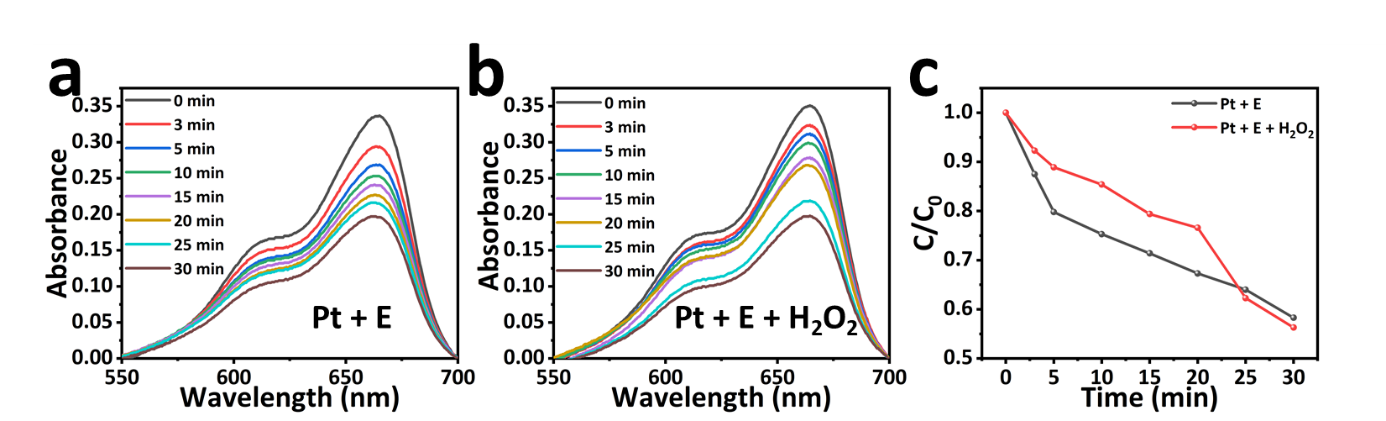


**Supplementary Figure 8.** (a) UV-vis absorption spectra of MB solutions degraded by Pt NPs under the 10 mHz AC field in the presence and absence of H_2_O_2_([Pt]: 200 µg mL^−1^, output current: 10mA, [MB]: 2.5 × 10^−5^ M, [H_2_O_2_]: 100 µM). (c) Degradation rates of MB in the presence of Pt NPs with or without H_2_O_2_.


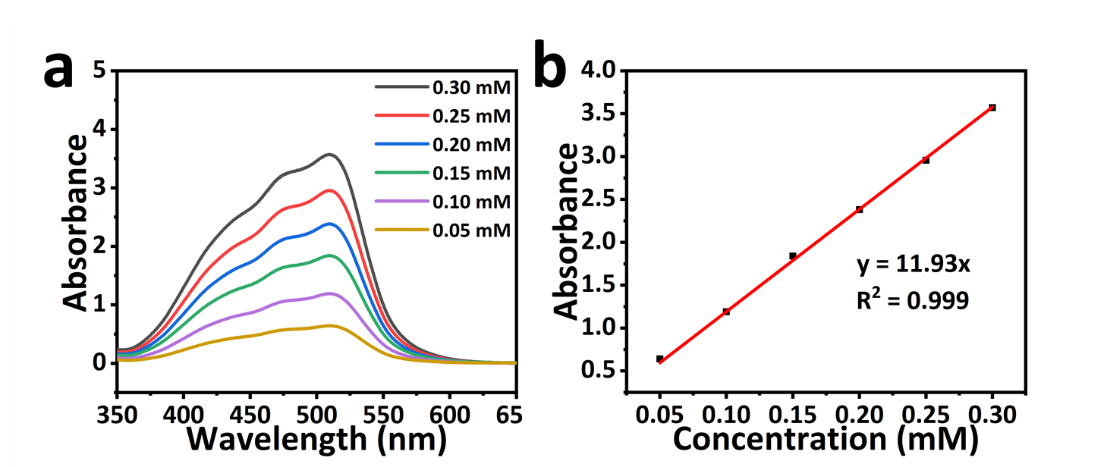


**Supplementary Figure 9.** (a) UV–vis absorbance spectra of 1,10-phenanthroline solutions with different Fe^2+^ concentrations, and (b) the relationship between the optical absorbance at 511 nm and the concentration of 1,10-phenanthroline solutions.


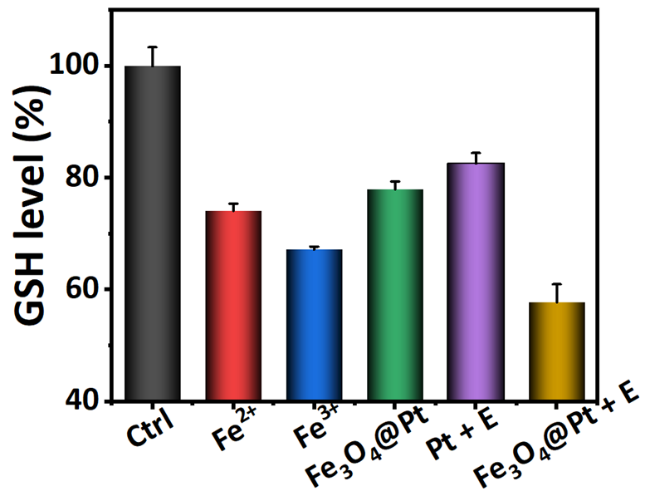


**Supplementary Figure 10.** Relative intracellular GSH in 4T1 cells with different treatments. ([Fe_3_O_4_]: 200 µg mL^−1^; electric field: square wave AC field; output current: 5mA, time: 10min).


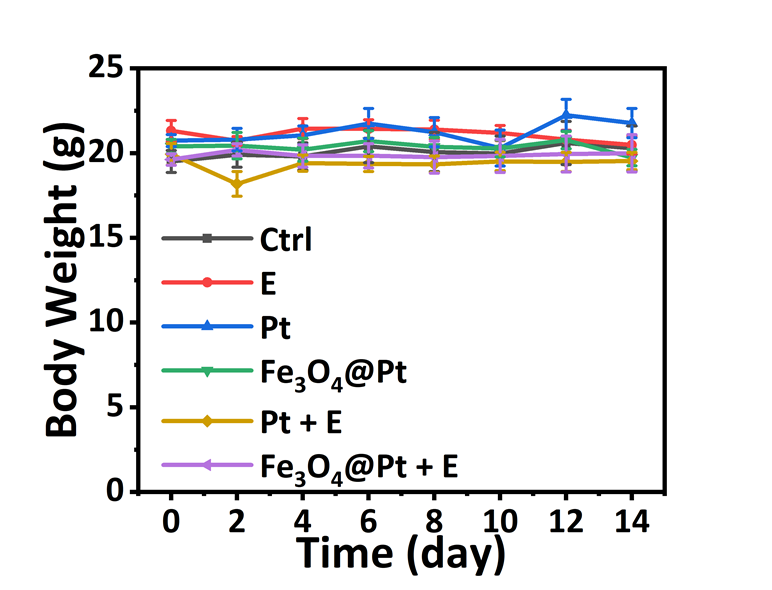


**Supplementary Figure 11.** Average body weights of mice after different treatments.
